# Supplementary material for: The Anti-Cancer Drug Dabrafenib Is a Potent Activator of the Human Pregnane X Receptor
Source: Cells. 2020 Jul 8;9(7):1641. doi: 10.3390/cells9071641 (PMC7407672; doi:10.3390/cells9071641)
Supplement: Supplementary file 1 [file cells-09-01641-s001.pdf]

SUPPLEMENTARY MATERIALS

Supplementary Table S1. List of reporter cell lines used in the study

Reporter cell line	Nuclear receptor	Reporter gene
HG5LN		GAL4RE$_5$-βGLOBIN-Luciferase
HG5LN GAL4-hPXR	GAL4-hPXR	GAL4RE$_5$-βGLOBIN-Luciferase
HG5LN GAL4-mPXR	GAL4-mPXR	GAL4RE$_5$-βGLOBIN-Luciferase
HG5LN GAL4-zfPXR	GAL4-zfPXR	GAL4RE$_5$-βGLOBIN-Luciferase
HG5LN GAL4-hCAR	GAL4-hCAR	GAL4RE$_5$-βGLOBIN-Luciferase
HG5LN GAL4-hCAR APYLT	GAL4-hCAR APYLT	GAL4RE$_5$-βGLOBIN-Luciferase
LS174T hPXR	hPXR	CYP3A4-Luciferase
HEPG2 hPXR	hPXR	CYP3A4-Luciferase
22RV1 hPXR	hPXR	CYP3A4-Luciferase

Supplementary Table S2. List of livers

Liver	Sex	Age	Pathology
FT438	Female	70	Cholangiocarcinoma
FT439	Male	73	Donor organ
FT440	Male	76	Metastasis of cholic adenocarcinoma

Supplementary Table S3. List of primers used in RT-PCR experiments

Gene	Forward	Reverse
CYP2B6	CCTTTTGGGAAACCTTCTGC	ACTCCACACAGCATGACCAC
CYP3A4	GCCTGGTGCTCCTCTATCTA	GGCTGTTGACCATCATAAAAG
ABCG2	CATGTACTGGCGAAGAATATT	CACGTGATTCTTCCACAAGCC
FGF-19	AGATCAAGGCAGTCGCTCTG	AAAGCACAGTCTTCCTCCGA
hPXR	GCAGGAGCAATTCGCCATT	TCGGTGAGCATAGCCATGATC
GAPDH *	GAGTCAACGGATTTGGTCGT	GACAAGCTTCCCGTTCTCAG
RLP0*	TCGACAATGGCAGCATCTAC	GCCTTGACCTTTTCAGCAAG

* Housekeeping genes

Supplementary Table S4.

	**HG5LN GAL4-HPXR**		**LS174T-PXR**		**HEPG2-PXR**		**22RV1-PXR**	
	% max act	EC_{50} (nM)	% max act	EC_{50} (nM)	% max act	EC_{50} (nM)	% max act	EC_{50} (nM)
**DMSO**	19.1		7.2		8.4		26.8	
**SR12813**	100	239 ± 109	100	434 ± 81	100	184.7 ± 11.7	100	125 ± 34
**Dabrafenib**	98.3	82 ± 13.9	96.6	110 ± 0.18	76.9	97.6 30.9	85.5	98.4 ± 42

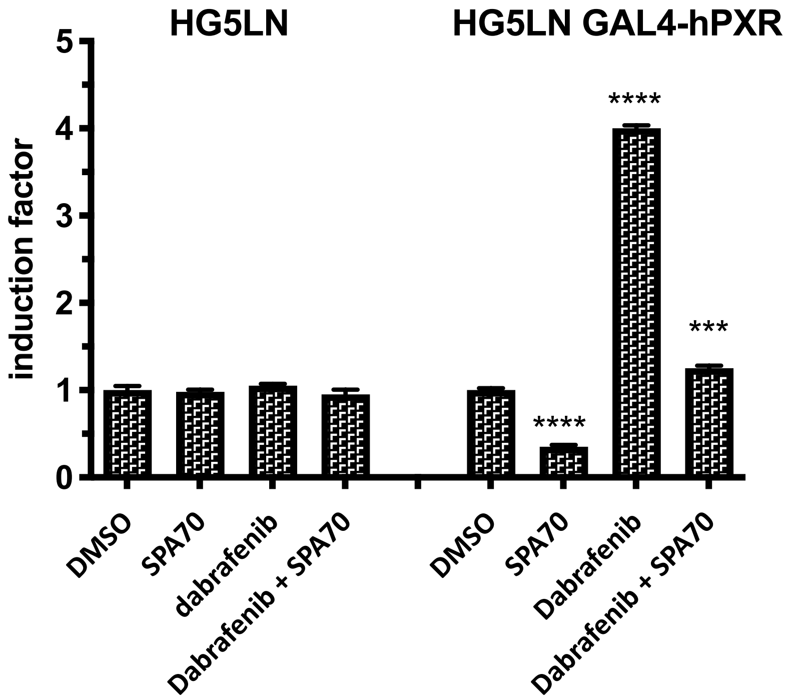

Supplementary Figure S1: Effects of dabrafenib on the activation of hPXR as measured by luciferase reporter assay. Activation has been measured in HG5LN control cells or HG5LN cells expressing human PXR (hPXR) treated by DMSO (0.1%), SPA70 3 µM, dabrafenib 0.3 µM and dabrafenib 0.3 µM + SPA70 3 µM for 24 hours. Results are expressed as fold induction as compared to control. Data are the mean ± sd of three independent experiments. **** $P < 0.0001$, *** $P < 0.001$ (Student's t-test) compared to DMSO treated cells.

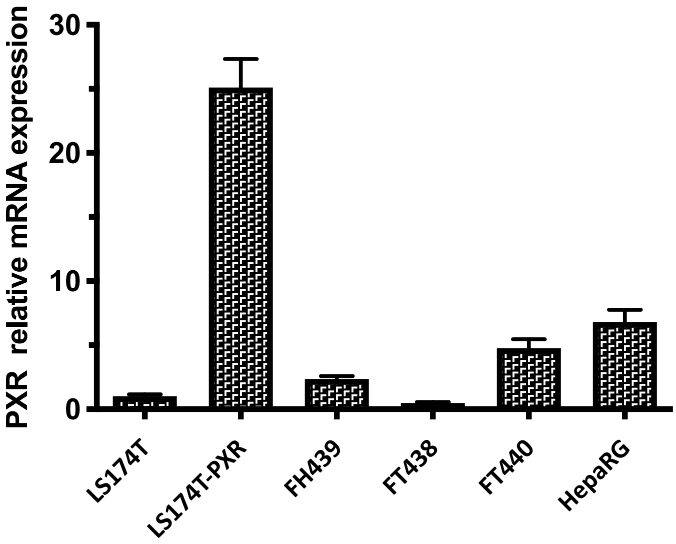

Supplementary Figure S 2: hPXR expression in LS174T, LS174T-PXR, differentiated HepaRG cells and fresh human hepatocytes. RT-qPCR of PXR mRNA expression were performed in LS174T, LS174T hPXR and differentiated HepaRG cells and human primary hepatocytes (three independent donors: FT438, FH439 and FH440). Results were obtained from three independent experiments performed in duplicate. The relative gene expression levels were normalized using GAPDH content.

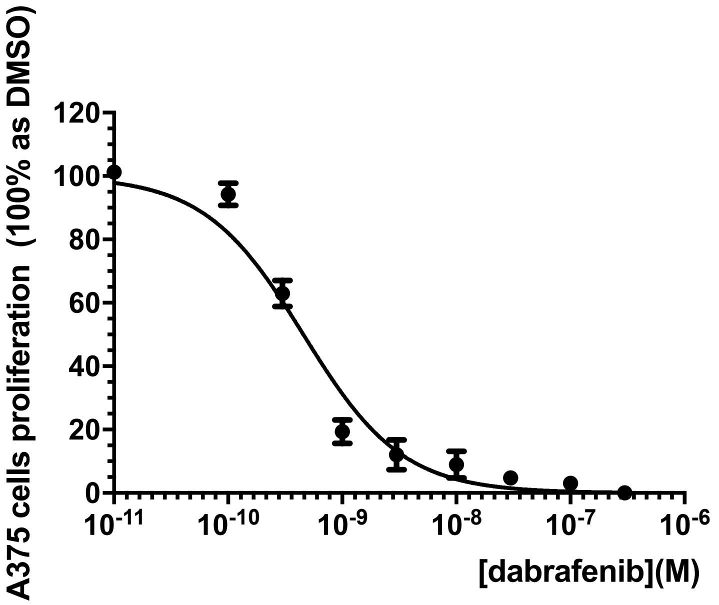

Supplementary Figure S3: Effects of dabrafenib on the inhibition of the proliferation of the $BRAF^{v600}$ mutated melanoma cell line A-375. A375 cells were treated with DMSO (0,1%) or increasing concentrations of dabrafenib for 4 days and cell growth was measured using MTT assay. Results are expressed as percentage of proliferation as compared to DMSO treated cells. Data are the mean ± sd of three independent experiments.
